# Intestinal Ultrasonographic Measurements in Cats Diagnosed with Lymphoplasmacytic Enteritis and Low-Grade T-Cell Lymphoma Based on Either Histology/Immunohistochemistry or Clonality Testing—And Assessment of the Effects of Therapy on Wall Layering After 3 and 6 Months of Treatment

**DOI:** 10.3390/ani15111518

**Published:** 2025-05-22

**Authors:** Laura Beatrice, Junwei Föhr, Paula Grest, Maja Ruetten, Manfred Henrich, Simona Vincenti, Karolin Campbell, Peter Hendrik Kook

**Affiliations:** 1 Clinic for Small Animal Internal Medicine, Vetsuisse Faculty, University of Zurich, 8057 Zurich, Switzerland; 2 Institute of Veterinary Pathology, Vetsuisse Faculty, University of Zurich, 8057 Zurich, Switzerland; 3 Pathovet AG, 8317 Tagelswangen, Switzerland; 4 Institute of Veterinary Pathology, Justus-Liebig University Giessen, 35392 Giessen, Germany; 5 Clinic for Small Animal Surgery, University of Zurich, 8057 Zurich, Switzerland; 6 Clinic of Diagnostic Imaging, Vetsuisse Faculty, University of Zurich, 8057 Zurich, Switzerland

**Keywords:** cat, enteritis, enteropathy, gastrointestinal, low-grade intestinal lymphoma, ultrasonography, small cell

## Abstract

It is unknown whether intestinal ultrasonographic measurements differ between lymphoplasmacytic enteritis (LPE) and low-grade intestinal T-cell lymphoma (LGITL) in cats if the diagnosis is based either on histology/immunohistochemistry (IHC) or on clonality assay results. The effects of treatment on intestinal ultrasonographic measurements are also unknown. Therefore, we prospectively compared small intestinal wall layering between cats with LPE and LGITL and investigated whether there were differences between the groups when the diagnostic gold standard was either histology/IHC or clonality testing. We evaluated the effects of standardized treatment in a subset of cats. The thicknesses of the total wall, mucosa, muscularis, and submucosa were measured in the duodenum, jejunum, and ileum, and ratios (muscularis to submucosa, muscularis to total wall thickness) were calculated. The thickness of the largest mesenteric lymph nodes was also determined. Ultrasonographic measurements from duodenal and jejunal segments were grouped together, and ileal segments were assessed separately. Sixteen cats with standardized full-thickness biopsies from the stomach, duodenum, jejunum, and ileum were included. Samples for clonality testing were fresh-frozen and analyzed later, and the standardized treatment was based on histologic/IHC diagnoses. Ultrasonographic measurements were compared between LPE and LGITL when diagnoses were either based on histology/IHC or clonality testing using a linear mixed model. Repeated ultrasonographic measurements of segments were available for seven cats after 12 weeks (five LPE, two LGITL) and five cats after 24 weeks (three LPE, two LGITL) of standardized treatment. We found that none of the ultrasonographic measurements differed between LPE and LGITL regardless of the diagnostic gold standard used. During treatment, only the ratio of lamina muscularis thickness to total wall thickness decreased significantly in LPE cats after 12 and 24 weeks compared to baseline. In conclusion, the herein evaluated ultrasonographic variables did not differ between LPE and LGITL and the diagnostic gold standard used had no influence on the results. The detected change over time during treatment in LPE cats requires further study.

## Simple Summary

It is unclear whether ultrasonographic examinations of the small intestines differ between cats with chronic enteritis and lymphoma if the diagnosis is made either histologically or based on lymphocyte clonality testing. Effects of treatment on ultrasound results have also never been assessed. We compared wall layer thicknesses between cats with enteritis and lymphoma diagnosed either with histology or clonality testing and also examined the effects of therapies in a subset of cats. Wall layering was measured in the duodenum, jejunum, and ileum, and ratios of individual layers were calculated. The thickness of the largest abdominal lymph node was also determined. Sixteen cats with surgical biopsies from stomach, duodenum, jejunum, and ileum were included. Medical treatments were based on histologic diagnoses. Ultrasonographic measurements were compared between enteritis and lymphoma when final diagnoses were either based on histology or clonality testing. The same ultrasonographic measurements were available for seven cats after 12 weeks and five cats after 24 weeks of medical treatment. No measurement differed between enteritis and lymphoma regardless of the diagnostic gold standard used. During treatment, only the ratio of muscular layer thickness to total wall thickness decreased significantly in cats with enteritis after 12 and 24 weeks compared to baseline. The change over time during treatment in cats with enteritis warrants further study.

## 1. Introduction

Feline chronic enteropathy is common in older cats that present with a combination of vomiting, diarrhea, anorexia, and weight loss. Within chronic enteropathy in cats, lymphoplasmacytic enteritis (LPE) and low-grade intestinal T-cell lymphoma (LGITL, also referred to as small cell lymphoma) are the two largest disease groups, while eosinophilic enteritis is less common [1,2,3]. LPE is the most common histologic finding in cats with chronic enteritis, and is often called inflammatory bowel disease in reference to humans. Both diseases, LPE and LGITL, are clinically indistinguishable [1,2].

Both diseases are often segmentally found in cats, and thus a definitive diagnosis usually requires biopsies from all small bowel segments [1,2,4,5,6].

Abdominal ultrasonography is routinely used to examine the gastrointestinal tract of cats presenting with chronic gastrointestinal clinical signs. While intestinal ultrasonography has a high positive predictive value for detecting histologic lesions, it cannot differentiate LPE from LGITL [7,8], and even experienced practitioners may find it challenging to differentiate between normal variations and pathological changes, further complicating interpretation [9]. Besides total wall thickness [8,10], a pattern of lamina muscularis thickening has been recognized in cats with lymphoma and cats with LPE [11,12]. While it has initially been thought that thickening of the lamina muscularis is more common in cats with LGITL [11], a follow-up study that compared individual US measurements to corresponding anatomical diagnoses found no difference in 14 cats with LPE (n = 6 cats) and LGITL (n = 8 cats) [12]. A more recent study comparing 22 cats with LPE to 22 cats with LGITL also found no differences in the thickness of the lamina muscularis between disease groups [13].

Routine work-up for FCE usually includes the collection of intestinal biopsies and the histopathologic evaluation of hematoxylin-and-eosin-stained sections [1,4]. There is controversy as to what constitutes the gold standard for differentiating LPE from LGITL in cats. Immunohistochemistry (IHC) tests with stains specific for T and B cells, as well as lymphocyte clonality assays, have been considered state of the art by some authors [4,5,14,15].

However, the specificity of clonality tests has recently been questioned [4,16,17].

Oftentimes there are conflicting results between histology/IHC and clonality testing [4], and clinicians are uncertain about the final diagnosis. Accurate diagnosis helps to optimize treatment, as cats with LPE are usually treated with prednisolone alone, while cats with LGITL receive chlorambucil in addition to prednisolone. Vitamin B12 can be low in both diseases and is therefore usually routinely supplemented [1,2]. At this time, a combination of histopathology and immunohistochemistry is increasingly regarded as the gold-standard test [1,2,4]. Because of the current uncertainty about the sensitivity and specificity of the aforementioned diagnostic modalities, we aimed to evaluate if standardized measurements from an intestinal ultrasonographic assessment would correlate better with a diagnosis based on histology/IHC or with a diagnosis based on clonality testing. Moreover, because no follow-up studies evaluating the effect of therapy on intestinal US markers exist in cats with FCE, our second aim was to examine if ultrasonographic measurements would change over time in cats receiving standard treatment for their disease.

## 2. Materials and Methods

This prospective study was conducted at the Clinic for Small Animal Internal Medicine, Vetsuisse faculty, University of Zurich, between 01/2015 and 02/2016. Written owner consent was obtained for each cat before enrollment into the study.

### 2.1. Case Selection

Cats examined at the Clinic for Small Animal Internal Medicine, University of Zurich, with clinical signs of chronic gastrointestinal tract disease (e.g., vomiting, diarrhea, inappetence, or weight loss) were eligible for enrollment in the study if the results of a complete blood count with serum biochemistry, including lipase activity [18], serum thyroxin measurement, trypsin-like immunoreactivity, and cobalamin concentrations; fecal examination; and abdominal ultrasonographic examination indicated enteropathy as the most likely cause for their clinical signs. Cats with systemic diseases (e.g., neoplastic disease), chronic illnesses (e.g., kidney disease, IRIS stage > 2, heart disease) or laboratory abnormalities that were deemed to be clinically relevant were excluded from the study.

### 2.2. Ultrasonography and Analyses

**Figure 1:**
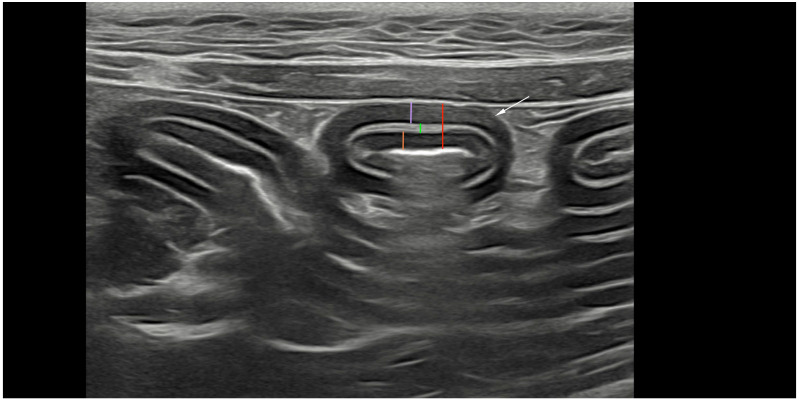
Ultrasonographic transversal image of the jejunum (white arrow; 8–12 MHz linear transducer). The red marking indicates the total wall thickness, the purple marking the lamina muscularis, the green marking the submucosa, and the orange marking the mucosa.

All abdominal ultrasonographic examinations were performed with the patient in dorsal recumbency. Representative ultrasonographic images were obtained using an 8–12 MHz linear transducer or curvilinear 5–8 MHz transducer (ATL 5000; Philips, Zofingen, Switzerland). All cats were fasted for a minimum of 8 h and bowel loops were free of gas. Three images of each bowel segment (duodenum, jejunum, and ileum) were obtained in transverse and sagittal planes, as described by Daniaux et al. [12]. At least three images were obtained of the largest mesenteric lymph nodes in the sagittal plane, and echogenicity and echotexture were recorded. We ultimately decided to only use the transverse plane for the ultrasonographic measurements of the intestinal wall and layer thickness based on the reported limitations of the longitudinal plane. Oblique measurements of the bowel cause errors of either the overestimation or underestimation of wall thickness [19]. Three full-thickness measurements of the duodenal and jejunal walls from the mucosa to the serosa of one wall, as well as measurements of the thickness of each layer (mucosa, submucosa, and muscularis) were performed (Figure 1). The maximal and minimal thicknesses of the ileal submucosa were measured in order to account for natural invaginations. Mesenteric lymph nodes were evaluated for maximal thickness, which was defined as the measurement perpendicular to the long axis of the largest lymph node. For all anatomical locations, the mean value of three measurements was used. The same ultrasonographic examinations were repeated after 12 and 24 weeks of standardized treatment, and the same measurements recorded at baseline were obtained after 12 and 24 weeks.

### 2.3. Sample Collection and Histopathologic Assessment

**Figure 2:**
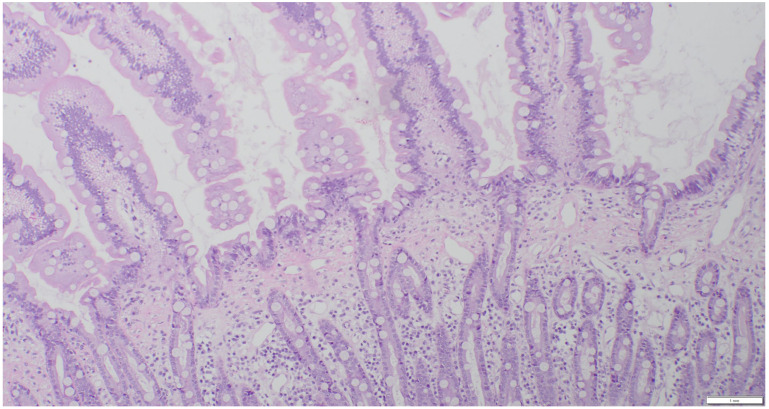
Hematoxylin and eosin stain (HE), 10× magnification. Jejunal biopsy of a cat with LPE. The intestinal villi are shortened, and slightly increased amounts of lymphocytes and plasma cells are present in the lamina propria.

All cats underwent an exploratory celiotomy with full-thickness biopsies of the stomach, duodenum, jejunum, ileum, and liver, and biopsy of the largest mesenteric lymph nodes. Biopsies were divided for routine histopathology and immunohistochemistry tests, as well as clonality testing. One half was placed in buffered formalin 10% for histopathology; the other half was snap-frozen at −80 °C for later clonality testing. All histopathologic examinations were performed by two experienced board-certified pathologists according to the WSAVA histopathologic scoring system and a consensus diagnosis was made after discussing the cases [20,21]. A diagnosis of LPE was made when mixed lymphoid and plasmacytic inflammatory infiltrates and occasional neutrophils were present (Figure 2).

**Figure 3:**
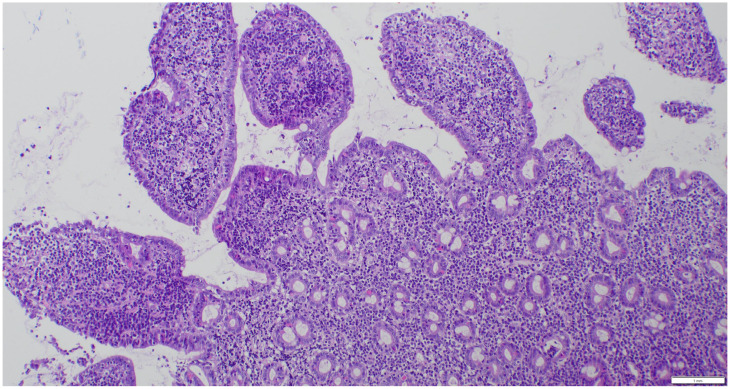
HE stain, 10× magnification. Diffuse massive infiltration of the lamina propria with additional small nests of epithelial lymphocytes.

**Figure 4:**
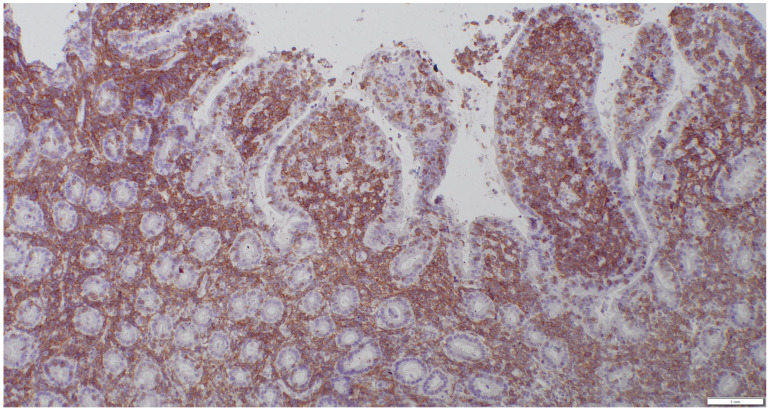
CD3 stain, 10× magnification. Same biopsy as in Figure 2; diffuse infiltration of CD3-positive lymphocytes.

**Figure 5:**
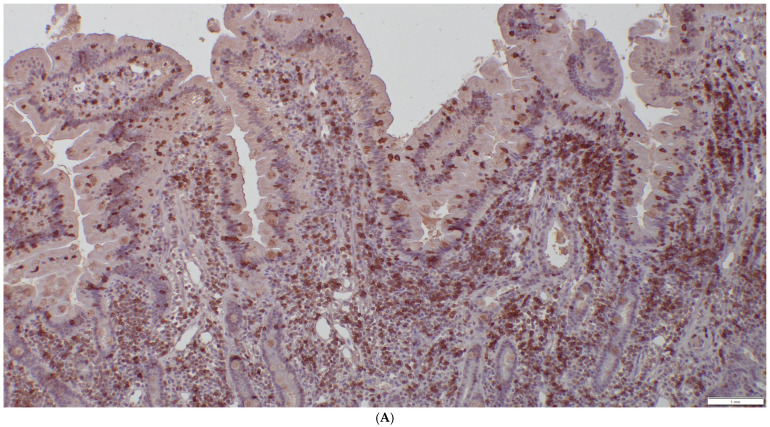

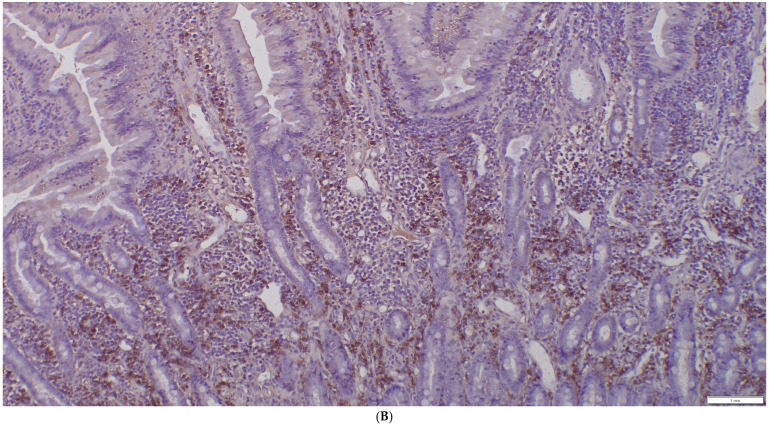
(**A**) CD3 stain, 10× magnification. Immunohistochemistry for CD3 shows approximately 50% of lymphocytes staining positive for CD3. (**B**) CD20 stain, 10× magnification. Immunohistochemistry for CD20 of the same biopsy as shown in (**A**) shows approximately 50% of lymphocytes staining positive for CD20.

Lymphomas were classified according to the WHO classification [22]. Assessment included mucosal versus transmural infiltration, the presence of epitheliotropism, cell size, and the pattern of lamina propria infiltration. A morphologic diagnosis of low-grade intestinal lymphoma was made when a moderate-to-marked increase in monomorphic small lymphoid cells was observed within the lamina propria or lamina epithelialis, extending beyond the mucosa and forming intraepithelial nests (Figure 3). Large cell lymphoma was diagnosed when dense proliferations of large lymphoid cells were present. Lymphoma was further classified as T-cell, B-cell, or non-T non-B cell lymphoma. Lymphocytes were assessed for CD3 (mouse/monoclonal, DAKO, M725401) and CD20 (rabbit/polyclonal, NeoMarkers RB-9013-P) positivity. Cases negative for both CD3 and CD20 were analyzed for the expression of CD79a (mouse/monoclonal, DAKO, M705101) because CD20 and CD79 are expressed sequentially and are not universally expressed across all stages of B-lymphocyte maturation [23]. A dense monomorphic infiltrate of T cells was suggestive of lymphoma (Figure 4), while a mixed, polymorphic infiltrate of T cells and B cells suggested LPE (Figure 5A,B).

### 2.4. Clonality Testing

Fresh-frozen biopsies were sent on dry ice to an external laboratory (University of Giessen, Germany) for clonality testing. All examinations were performed by a board-certified pathologist with expertise in clonality testing who was blinded to the clinical status of the cats. Each sample was amplified in 6 multiplex reactions according to Mochizuki et al. (2011) and Mochizuki et al. (2012), with 6 different primer sets [24,25]: Multiplex FR1 (V1F1, V3F1, JR1, JR2, JR3, JR4, and JR5), Multiplex FR3 (V1F2, V3F4, JR1, JR2, JR3, JR4, and JR5), V3F2 (V3F2, JR1, JR2, JR3, JR4, and JR5), V3F3 (V3F3, JR1, JR2, JR3, JR4, and JR5), fTCRγ-J1 (ftcrgv1–2, ftcrgv3, ftcrgv4, ftcrgv5, ftcrgj1), and fTCRγ-J2 (ftcrgv1–2, ftcrgv3, ftcrgv4, ftcrgv5, ftcrgj2) [24,25].

Capillary electrophoresis was performed on an ABI Prism^®^ 310 Genetic Analyzer (Applied Biosystems^®^ by life technologies, Darmstadt, Germany) using the Bioline MyTaq™ HS Mix (Bioline GmbH, Luckenwalde, Germany) according to the protocols of Mochizuki [21,22]. Briefly, the reaction mix consisted of 7.5 μL MyTaq HS Mix, 2x, primer mix, and 30 ng sample DNA. The reaction volume was adjusted to 15 μL with sterile water. Each reaction was performed in duplicate, and for each PCR run a clonal, a polyclonal, and a non-template control were included.

Amplification consisted of an initial activation step (95 °C, 15 min) followed by 40 cycles of denaturation (95 °C, 30 s), annealing (68 °C, 90 s), and elongation (72 °C, 30 s). A terminal elongation step at 72 °C for 30 min was added to harmonize “plus A” artifacts. Samples were diluted post-PCR 1:15 in water. The diluted product was denatured in formamide (Hi-DiTm Formamide, Applied Biosystems^®^ by life technologies, Darmstadt, Germany) and mixed with LIZ size standard (GeneScan™ 500 LIZ™ dye Size Standard, Applied Biosystems^®^ by life technologies, Darmstadt, Germany) at 95 °C for 5 min, followed by 10 min incubation on ice. Injection was performed at 15.000 V for 5 s. The temperature during electrophoresis was 60 °C.

Analysis of the electropherograms was performed using the Peak Scanner™ Software (version 1.0, Applied Biosystems^®^ by life technologies, Darmstadt, Germany). Clonality was considered if one or two discernable peaks were visible and reproducible in the replicates. Pseudoclonality was considered if peaks were not reproducible, and the polyclonal pattern was defined as multiple peaks of PCR products spread over the expected size range, ideally in a Gaussian curve-like distribution.

### 2.5. Treatment

Cats with LPE were treated with prednisolone (median 1.4 mg/kg, range 1.2–2 mg/kg PO, once daily) for 24 weeks. Cats with LGITL were treated with prednisolone (median 1.5 mg/kg, range 1.3–1.9 mg/kg PO, once daily) and chlorambucil. Chlorambucil was dosed according to body weight: cats weighing < 4 kg received 2 mg PO given every 3rd day; cats > 4 kg received 2 mg every 2nd day for 2 weeks, then 2 mg every 3rd day. As clonality testing results were not immediately available, all subsequent ultrasonographic measurements during treatment (t12, t24) are based on the diagnostic gold standard of histology/IHC.

### 2.6. Statistical Evaluation

We evaluated differences in the mean thickness of the intestinal segments among small intestines diagnosed with LPE and LGITL according to the theoretical gold-standard histopathology and IHC, and according to the theoretical gold-standard clonality testing. Measurements from the duodenum and jejunum were grouped together (duodenum and jejunum), since previous studies showed no significant differences in layering between the two [26]. The ratio of the mean thickness of the lamina muscularis to the mean thickness of the submucosa [10] and the ratio of the mean thickness of the lamina muscularis to total wall thickness were calculated. We tested for differences in the thickness of the intestinal layers (mucosa, submucosa, muscularis), for differences in the total wall thickness, and differences in the above-mentioned ratios of the duodenum and jejunum (combined), as well as of the ileum, with a linear mixed model. Analysis was undertaken in R [27], using the lme4 package [28]. Differences in the size of the mesenteric lymph nodes of cats diagnosed with LPE or LGITL according to Histo + IHC and clonality testing were also analyzed using a linear mixed model. A repeated measures ANOVA and Tukey’s multiple comparison test were used to compare the results of the ultrasonographic measurements of the duodenum and jejunum combined, as well as the ileum between pre-treatment (t0) and 12 (t12) and 24 (t24) weeks after the standardized treatment for LPE and LGITL. For all analyses, a significance level of 0.05 was used in hypothesis testing. Post hoc analysis (eg qq plots) indicated that suitable statistical models were used for the analysis.

## 3. Results

### 3.1. Study Population

Eighteen cats were initially included in the study. In 2/18 cats, large cell lymphoma was diagnosed. In one case, gastric large B-cell lymphoma was diagnosed, characterized by dense sheets of CD20-positive large lymphocytes in the gastric mucosa. In the other case, proliferating large CD3-positive lymphoid cells were detected in all examined intestinal segments. These cats were excluded, ultimately leaving 16 cats in the study. The 16 cats had a median age of 10 years (range, 7–15), median body weight of 4.2 kg (range 2.1–6), and median body condition score of 4 out of 9 (range, 1–7). There were nine spayed female cats and seven neutered male cats. Breeds included domestic shorthair (n = 7), Norwegian Forest Cat (n = 2), Persian (n = 2), domestic longhair (n = 1), Oriental shorthair (n = 1), British shorthair (n = 1), domestic shorthair–Turkish angora mix (n = 1), and Neva Masquarade (n = 1).

### 3.2. Results of Histology/IHC and Clonality Testing

Based on histology/IHC, 11 cats were diagnosed with LPE and 5 cats with LGITL. In cats with LGITL, the histologic examination of the intestinal biopsies revealed dense, monomorphic infiltrates of small CD3-positive lymphocytes within the lamina propria, with variable extension into the epithelium and, in some cases, into the submucosal and muscular layers. Based on clonality testing, 11 cats were diagnosed with LPE and 5 cats with LGITL. Discordant results between histology/IHC and clonality testing were found in 6/16 (38%) cats, while 10 cats (62%) had concordant results. According to the clonality testing results, three cats would have been re-classified having LGITL and three as having LPE, which means eight cats with LPE had concordant results, while two cats with LGITL had concordant results, and depending on what is regarded as the diagnostic gold standard, they may not have received optimal treatment. Thus, we briefly review these cases below.

#### 3.2.1. Overview of the Course of Disease in Cats with Discordant Results

Of the three cats diagnosed with LPE via histology/IHC and LGITL based on clonality testing, one cat had persistent diarrhea and continued to gradually lose weight despite prednisolone treatment. Chlorambucil was added to the treatment 486 days after the biopsies, and the cat died 29 days later. The second cat diagnosed with LPE via histology/IHC and LGITL upon clonality testing performed clinically well with tapering doses of prednisolone, and ultimately died after 1403 day because of chronic kidney disease. The third cat diagnosed with LPE via histology/IHC and LGITL upon clonality testing had persistent diarrhea despite treatment with prednisolone, did not respond to chlorambucil, and became anemic (hematocrit 19%) 69 days after the biopsies. The cat was euthanized.

Of the three cats diagnosed with LGITL via histology/IHC and LPE upon clonality testing, one cat performed well clinically for 1587 days with combined prednisolone/chlorambucil treatment and regular cobalamin supplementation. The second cat diagnosed with LGITL via histology/IHC and LPE upon clonality testing performed well with combined prednisolone/chlorambucil treatment for 967 days until vomitus re-occurred. A gastric mass was detected ultrasonographically and cytologically diagnosed as large cell lymphoma, and the cat died after 987 days. The third cat diagnosed with LGITL via histology/IHC and LPE upon clonality testing had persistent diarrhea throughout the treatment that could not be controlled with additional treatments (probiotics, antibiotics, fecal microbiome transplantation) given after week 24, and died after 807 days. Postmortem histologic examinations of multiple small bowel segments showed LGITL in all biopsies; repeated clonality testing was not performed.

#### 3.2.2. Histology/IHC and Clonality Testing of Sampled Lymph Nodes

Only one cat with LGITL had dense, monomorphic infiltrates of small CD3-positive lymphocytes in the sampled lymph nodes and clonal results upon clonality testing. All other cats had diagnoses of reactive hyperplasia based on histology/IHC together with polyclonal results upon clonality testing. Both cats with large cell lymphoma also had histological and immunohistochemical evidence of large cell lymphoma in the lymph nodes, as well as clonal results in the clonality assay.

### 3.3. Ultrasonographic Measurements

For the evaluation of intestinal wall layer measurements at baseline, 32 measurements were available for the duodenum and jejunum taken together, 15 for the ileum, and 16 for the mesenteric lymph nodes. Seven cats were available for assessment of the effects of standardized treatment. Five cats with LPE (10 measurements of duodenum and jejunum) were available at 12 weeks, and three (6 measurements of duodenum and jejunum) were available at 24 weeks. Two cats with LGITL were available for follow-up measurements at 12 and 24 weeks (four measurements of duodenum and jejunum each).

In the duodenum, jejunum, and ileum, the thickness of the mucosa, submucosa, and muscularis, total wall thickness, the ratio of the mean thickness of the lamina muscularis to the mean thickness of the submucosa, and the ratio of the mean thickness of the lamina muscularis to the total wall thickness were not statistically different between LPE and LGITL, irrespective of the applied diagnostic gold standard. Table 1a,b show the results for all measurements.

Assessing intestinal segments according to the cat’s final diagnosis and not per segment diagnosis also did not show any statistical differences between LPE and LGITL, irrespective of the applied diagnostic gold standard. The size of the mesenteric lymph nodes was not statistically different between LPE and LGITL, irrespective of the applied diagnostic gold standard (Table 2).

**Figure 6:**
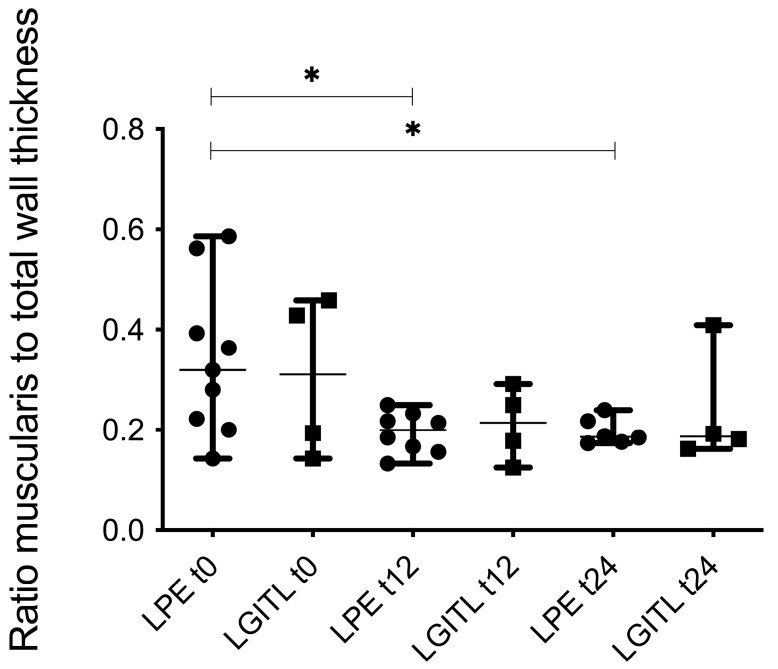
The ratio of lamina muscularis to total wall thickness in the duodenum and jejunum decreased significantly in cats with LPE during treatment with prednisolone. Significant differences (*) were found after 12 weeks of treatment (t12) (*p* = 0.0148), as well as 24 weeks of treatment (t24) (*p* = 0.0145) compared to the time of diagnosis (t0). There was no significant difference between t12 and t24.

When assessing ultrasonographic measurements over time during treatment, the ratio of the mean thickness of the lamina muscularis to the total wall thickness decreased significantly over time in cats diagnosed with LPE in the duodenum and jejunum group. A significant decrease was found for this marker between t0 and t12 (*p* = 0.0148) and between t0 and t 24 (*p* = 0.0145) (Figure 6).

No other statistically significant change was observed over time in terms of the thickness of the mucosa, submucosa, and muscularis, the total wall thickness, or the ratio of the mean thickness of the lamina muscularis to the mean thickness of the submucosa.

Scant amounts of abdominal free fluid were found in 4/16 (25%) cats. Two of these four cats had discordant results with the applied diagnostic gold standard (one cat LPE via histology/IHC, LGITL upon clonality testing; one cat LGITL via histology/IHC and LPE upon clonality testing). Abdominal effusion was present in one cat with LGITL and LPE each, based on both diagnostic gold standards. Table 3 gives an overview of the measurements during the treatment over time.

## 4. Discussion

The present study prospectively evaluated intestinal wall layering in cats with LPE and LGITL in this study. At the time the study was undertaken, there was uncertainty as to what constitutes the gold standard to differentiate LPE from LGITL in cats, and the aim was to evaluate if ultrasonographic measurements differ between LPE and LGITL when applying both of the discussed diagnostic gold standards to biopsies. The coexistence of neoplastic and inflammatory cells in the same area of the sample, or neoplastic populations characterized by nests and plaques emerging in an inflammatory background, can complicate histological classifications. Therefore, the aim was to investigate whether a pattern could be detected where thicknesses correlate better with clonality than histology/IHC. However, this was not the case in this study. Zwingenberger et al. showed that the lamina muscularis is thicker in lymphoma cases compared to LPE based on odds ratio calculations; however, their approach was different, as a lamina muscularis thickening was defined as a thickness > 50% of the submucosa [11]. Instead, our study used the ratio of lamina muscularis to the submucosa—similar to two follow-up studies [12,13]—but also found no differences. Also, the ratio of the muscularis to total wall thickness was not different between the groups. This had not been assessed at the time the study was undertaken. However, the same finding has recently also been published by Freiche et al. in a larger cohort of cats [13]. The thickness of the mucosal layer in the combined duodenal and jejunal samples was not different between the disease groups, irrespective of the chosen diagnostic gold standard. The results from a newer study where LGITL cats had significantly thicker jejunal mucosal thicknesses compared to LPE cats differ compared to our results [13]. However, when looking at the results, the actual measurements were very similar in both studies. The differences between the studies may have been due to our decision to group duodenal and jejunal samples together in order to increase statistical power. We undertook this approach because individual wall layering has been found to be the same in the duodenum and jejunum of healthy cats [26]. Since the average contribution of each intestinal layer to total wall thickness is the same in the duodenum and jejunum, and also the total thickness of both segments does not differ in healthy cats [26], we theorized that changes would be equally recognizable as the starting point was the same for both small bowel segments.

There was also no difference in the maximal thicknesses of the mesenteric lymph nodes between LPE and LGITL, irrespective of what gold standard was used. This is an original finding of our study. Daniaux and colleagues compared lymph node measurements between healthy cats and LGITL cases, not between LPE and LGITL [12]. In the largest study so far, jejunal lymph nodes were significantly thicker in cats with LGITL in comparison to cats with LPE [13]. In our study, LGITL cats—although not significantly different to LPE cats—had smaller lymph nodes when looking at the mean and median results. Previous studies reported the enlargement of mesenteric lymph nodes upon ultrasound in cats diagnosed with lymphoma and LPE based on different references for normal mesenteric lymph nodes [12,29]. Taking the reference of <5 mm in normal cats [29,30], our results show a mild mesenteric lymph node enlargement in cats with LPE but not LGITL when looking at the median results. However, a limitation in this regard is that it cannot be ascertained that the identical lymph nodes were examined ultrasonographically and histologically.

The present study used two different approaches when comparing the ultrasonographic measurement results between LPE and LGITL. Diagnoses from individual segments were compared with ultrasonographic measurements, as well as ultrasonographic measurements between cats with final patient diagnoses of LPE and LGITL. It is our belief that the segment approach is more accurate, as feline enteropathy is often characterized by segmental distribution in the small intestine [31], and therefore segments with a diagnosis of LPE would have been erroneously handled as LGITL if, for example, LGITL was only found in the ileum. Still, baseline data were also analyzed with a “per patient diagnosis”, because on the one hand precise ultrasonographic image sampling and histologic sampling at the same site within the bowel segment was not possible, and on the other hand, cats were treated according to the final histologic diagnosis, and we wanted to assess if the standardized treatments had a measurable impact on evaluated ultrasonographic variables. However, all our results demonstrate that none of the evaluated ultrasonographic measurements differ between feline LPE and LGITL, which confirms the results by Daniaux and colleagues [12].

When evaluating measurements over time during treatment, only one variable (the ratio of muscularis to total wall thickness) from the combined duodenal and jejunal samples significantly decreased during treatment in cats with LPE. This could not be shown for the LGITL group, as the small sample size hampered analyses in the LGITL group during treatment. The change in the ratio of muscularis to total wall thickness is interesting because all follow-up evaluations are based on patient diagnosis, not segment diagnosis. The change in this ratio implies that overall lamina muscularis thickening does diminish under prednisolone in cats with LPE, as the other explanation of increasing total wall thickness seems less likely and is also not apparent when looking at both measurements (lamina muscularis and total wall thickness) over time per group. Median values of lamina muscularis decreased, while total wall thicknesses did not.

The lacking significance for the same ratio in cats with LGITL (in addition to the small sample size), might suggest that small intestinal tissues densely infiltrated with neoplastic small lymphocytes are less likely to shrink under prednisolone/chlorambucil treatment. However, further studies with a larger population are clearly necessary to make definitive statements. Our results cannot be compared to the literature, as no study has ever evaluated intestinal ultrasonography during treatment in cats with CE. It would be of interest to examine if a decrease in the duodenal/jejunal lamina muscularis during treatment correlates with clinical variables such as appetite, fecal score, and most importantly body weight.

Our results also confirm previous findings that a muscularis-to-submucosa ratio > 1 is generally indicative of small intestinal disease [12], although this ratio did not differ between LPE and LGITL.

Our main limitation is the small sample size; 16 cats were included at baseline and 7 for follow-up. However, this is the very first report on ultrasonographic follow-up in cats treated for chronic enteropathy. Unfortunately, not all ultrasonographic measurements were available from all cats. The distribution of LPE–LGITL in our study was also shifted to more LPE cats. The other two studies that have assessed quantified wall layer thicknesses in cats with LPE and LGITL included 14 [12] and 44 cats [13], and both studies had a more balanced relationship between disease groups. Although cats with LPE and LGITL are common presentations in small animal practice, no standardized prospective studies larger than what has been discussed here have been published. The results of the most recent study suggested that the presence of abdominal fluid was more frequent in cats with LGITL compared to LPE, although the reason for this is currently unclear [13]. However, only scant amounts of abdominal free fluid were found in 4 of 16 cats, and this number was evenly distributed between disease groups.

## 5. Conclusions

Even if no major significant differences were found in this study, this does not mean that ultrasonographic examinations of the intestinal tract are without benefit. Ultrasonography will still be performed during diagnostic work up in order to assess the extent of extraintestinal disease and also to check for focal lesions or abnormal lymph nodes that might be suitable for fine needle aspiration. However, quantitating layers, as well as the ratios of the measured layers, does not seem to differentiate LPE from LGITL, and ultrasonographic markers did not correlate better with clonality testing diagnoses. Larger studies are needed, especially for the assessment of the clinical relevance of follow-up intestinal ultrasonography during treatment.

## Figures and Tables

**Table 1:** (**a**) Mean + SD thickness (mm) and median (range) (mm) of intestinal wall layers of the duodenum and jejunum of 16 cats diagnosed with lymphoplasmacytic enteritis (LPE) or low-grade intestinal T-cell lymphoma (LGITL) based on either histology and immunohistochemistry (Histo + IHC) or clonality testing. None of the ultrasonographic variables differed significantly between groups, regardless of which diagnostic gold standard was used (statistical significance level set at 0.05). (**b**) Mean ± SD thickness (mm) and median (range) (mm) of intestinal wall layers of ileum of 15 cats diagnosed with lymphoplasmacytic enteritis (LPE) or low-grade intestinal T-cell lymphoma (LGITL) based on either histology and immunohistochemistry (Histo + IHC) or clonality testing. None of the ultrasonographic variables differed significantly between groups, regardless of which diagnostic gold standard was used (statistical significance level set at 0.05).

(a)								
	Duodenum and Jejunum							
Diagnostic Method	Histo + IHC				Clonality Testing			
Diagnosis	n	LPE	n	LGITL	n	LPE	n	LGITL
Total Wall thickness	21	2.52 ± 0.50	10	2.65 ± 0.41	19	2.47 ± 0.44	12	2.68 ± 0.51
		2.50 (1.70–3.50)		2.75 (2.00–3.20)		2.40 (1.70–3.50)		2.75 (1.80–3.30)
Mucosa	21	1.34 ± 0.36	10	1.29 ± 0.40	19	1.34 ± 0.39	12	1.30 ± 0.35
		1.30 (0.60–2.10)		1.30 (0.80–2.00)		1.30 (0.60–2.00)		1.30 (0.80–2.10)
Submucosa	21	0.40 ± 0.12	10	0.53 ± 0.12	19	0.43 ± 0.13	12	0.44 ± 0.14
		0.40 (0.30–0.70)		0.55 (0.30–0.70)		0.40 (0.30–0.70)		0.40 (0.30–0.70)
Muscularis	21	0.72 ± 0.44	10	0.73 ± 0.33	19	0.66 ± 0.36	12	0.80 ± 0.45
		0.60 (0.30–1.80)		0.65 (0.40–1.40)		0.50 (0.30–1.70)		0.70 (0.30–1.80)
Muscularis: submucosa	21	1.80 ± 1.01	10	1.44 ± 0.74	19	1.66 ± 0.97	12	1.82 ± 0.98
		1.40 (1.00–4.50)		1.20 (0.67–3.00)		1.33 (0.67–4.25)		1.37 (1.00–4.50)
Muscularis: total wall thickness	21	0.27 ± 0.13	10	0.28 ± 0.12	19	0.26 ± 0.12	12	0.29 ± 0.12
		0.24 (0.13–0.59)		0.25 (0.14–0.46)		0.23 (0.13–0.59)		0.26 (0.13–0.56)
(**b**)								
	**Ileum**							
**Diagnostic Method**	**Histo + IHC**				**Clonality testing**			
**Diagnosis**	**n**	**LPE**	**n**	**LGITL**	**n**	**LPE**	**n**	**LGITL**
Total Wall thickness	11	3.25 ± 0.68	4	3.78 ± 0.80	10	3.31 ± 0.87	5	3.54 ± 0.26
		3.40 (2.00–4.20)		3.60 (3.00–4.90)		3.20 (2.00–4.90)		3.60 (3.20–3.90)
Mucosa	11	1.40 ± 0.26	4	1.83 ± 0.54	10	1.48 ± 0.45	5	1.58 ± 0.24
		1.40 (1.00–1.90)		1.80 (1.20–2.50)		1.35 (1.00–2.50)		1.60 (1.30–1.90)
Submucosa	11	0.59 ± 0.14	4	0.55 ± 0.06	10	0.61 ± 0.14	5	0.52 ± 0.08
		0.60 (0.40–0.80)		0.55 (0.50–0.60)		0.60 (0.40–0.80)		0.50 (0.40–0.60)
Muscularis	11	1.33 ± 0.56	4	1.28 ± 0.25	10	1.26 ± 0.50	5	1.42 ± 0.50
		1.20 (0.40–2.20)		1.25 (1.00–1.60)		1.25 (0.40–2.20)		1.20 (1.00–2.10)
Muscularis: submucosa	11	2.35 ± 1.05	4	2.31 ± 0.29	10	2.17 ± 1.06	5	2.68 ± 0.58
		2.40 (0.57–4.40)		2.28 (2.00–2.67)		2.17 (0.57–4.40)		2.50 (2.00–3.50)
Muscularis: total wall thickness	11	0.39 ± 0.10	4	0.34 ± 0.05	10	0.36 ± 0.09	5	0.40 ± 0.12
		0.38 (0.20–0.54)		0.35 (0.28–0.40)		0.36 (0.20–0.52)		0.38 (0.28–0.54)

**Table 2:** Mean ± SD thickness (mm) and median (range) (mm) of mesenteric lymph nodes of 16 cats diagnosed with lymphoplasmacytic enteritis (LPE) or low-grade intestinal T-cell lymphoma (LGITL) based on either histology and immunohistochemistry (Histo + IHC) or clonality testing. There were no significant differences between groups, regardless of which diagnostic gold standard was used.

Diagnostic Method	Histo + IHC				Clonality Testing			
	n	LPE	n	LGITL	n	LPE	n	LGITL
Thickness (mm) of mesenteric lymph nodes	11	5.84 ± 2.47	5	4.70 ± 1.64	11	5.79 ± 2.64	5	4.92 ± 1.34
		6.00 (2.00–10.00)		4.90 (2.50–7.00)		6.00 (2.00–10.00)		5.00 (3.00–6.80)

**Table 3:** Changes in mean ± SD (mm) and median (range) (mm) of intestinal wall layers of the duodenum and jejunum of cats diagnosed with lymphoplasmacytic enteritis (LPE) or low-grade intestinal T-cell lymphoma (LGITL) based on histology and immunohistochemistry (Histo + IHC) over time. Five cats with LPE (10 measurements of duodenum and jejunum) were available at 12 weeks, and three (6 measurements of duodenum and jejunum) at 24 weeks. Two cats with LGITL were available for follow-up measurements at 12 and 24 weeks (4 measurements of duodenum and jejunum each). Only the muscularis-to-full-thickness ratio differed significantly (*p* < 0.05) between baseline examination and 12 weeks (*p* = 0.0148) and baseline and 24 weeks (*p* = 0.0145) of treatment.

**Time Point**	**0 (Baseline Examination)**				**12th Week of Treatment**				**24th Week of Treatment**			
**Diagnosis**	**n**	**LPE**	**n**	**LGITL**	**n**	**LPE**	**n**	**LGITL**	**n**	**LPE**	**n**	**LGITL**
Total Wall thickness	10	2.60 ± 0.55 2.70 (1.70–3.30)	4	2.60 ± 0.44 2.60 (2.10–3.10)	10	2.63 ± 0.38 2.65 (2.00–3.20)	4	2.70 ± 0.38 2.60 (2.40–3.20)	6	2.52 ± 0.51 2.40 (1.80–3.20)	4	2.68 ± 0.71 2.40 (2.20–3.70)
Mucosa	10	1.22 ± 0.41 1.20 (0.60–2.00)	4	1.38 ± 0.61 1.35 (0.80–2.00)	10	1.70 ± 0.52 1.55 (1.10–2.80)	4	1.80 ± 0.39 1.75 (1.40–2.30)	6	1.52 ± 0.21 1.45 (1.30–1.90)	4	1.48 ± 0.55 1.35 (1.00–2.20)
Submucosa	10	0.43 ± 0.14 0.40 (0.30–0.70)	4	0.50 ± 0.14 0.55 (0.30–0.60)	10	0.40 ± 0.12 0.45 (0.20–0.50)	4	0.48 ± 0.10 0.45 (0.40–0.60)	6	0.40 ± 0.11 0.40 (0.30–0.60)	4	0.40 ± 0.08 0.40 (0.30–0.50)
Muscularis	10	0.94 ± 0.55 0.80 (0.30–1.80)	4	0.75 ± 0.31 0.75 (0.40–1.10)	10	0.51 ± 0.09 0.50 (0.40–0.70)	4	0.55 ± 0.13 0.55 (0.40–0.70)	6	0.48 ± 0.12 0.50 (0.30–0.60)	4	0.60 ± 0.22 0.55 (0.40–0.90)
Muscularis: submucosa	10	2.25 ± 1.35 1.67 (1.00–4.50)	4	1.66 ± 1.00 1.42 (0.80–3.00)	10	1.38 ± 0.44 1.33 (0.80–2.00)	4	1.16 ± 0.20 1.13 (1.00–1.40)	6	1.26 ± 0.43 1.13 (0.83–2.00)	4	1.52 ± 0.53 1.42 (1.00–2.25)
Muscularis: total wall thickness	10	0.34 ± 0.15 0.32 (0.18–0.59)	4	0.31 ± 0.16 0.31 (0.14–0.46)	10	0.20 ± 0.04 0.20 (0.13–0.25)	4	0.21 ± 0.07 0.21 (0.13–0.29)	6	0.19 ± 0.03 0.18 (0.17–0.24)	4	0.24 ± 0.12 0.19 (0.16–0.41)

## Data Availability

The data presented in this study are available on request from the corresponding author.
